# The Complete Mitochondrial Genome of Nearly Threatened Kwang‐Yang Asian Frog *Nanorana quadranus* (Anura: Dicroglossidae) and Its Phylogenetic Analyses

**DOI:** 10.1002/ece3.72877

**Published:** 2026-01-11

**Authors:** Jia Liu, Bin Zuo, Yan‐Bo Sun

**Affiliations:** ^1^ Key Laboratory of Pu‐Er Tea Science, Ministry of Education Yunnan Agricultural University Kunming China; ^2^ College of Science Yunnan Agricultural University Kunming China; ^3^ State Key Laboratory of Vegetation Structure, Functions and Construction, and Yunnan Key Laboratory of Biological Adaptation, Conservation and Utilization, School of Ecology and Environmental Science Yunnan University Kunming China; ^4^ Southwest United Graduate School Kunming China

**Keywords:** Mitogenome, *Nanorana quadranus*, Phylogenetic analysis, Qinghai‐Tibet plateau

## Abstract

*Nanorana quadranus* (Liu, Hu & Yang, 1960) is a high‐altitude amphibian species native to the Qinghai‐Tibet Plateau. It plays a vital role in alpine wetland ecosystems and is listed as near threatened (NT) in the IUCN Red List. Here, we present the first complete mitogenome of *N. quadranus* using the Illumina NovaSeq 6000 sequencing platform. The total length of the mitogenome is 20,173 bp (Accession No. PV546862). It encompasses 13 protein‐coding genes (PCGs), 21 transfer RNAs (tRNAs), 2 ribosomal RNAs (rRNAs), and one control region. Phylogenetic analysis revealed that *N. quadranus* forms a clade with *N. taihangnica* , supporting its taxonomic placement within the Dicroglossidae family. This study provides foundational genetic data for further research on the evolution and conservation of this species.

## Graphical abstract

We report the first complete mitochondrial genome of the near‐threatened frog *Nanorana quadranus* from the Qinghai‐Tibet Plateau. The 20,173 bp mitogenome includes 13 protein‐coding genes, 21 tRNAs, 2 rRNAs, and a control region. Phylogenetic analysis places *N. quadranus* in a clade with *N. taihangnica* , providing key genetic data for future studies on evolution and conservation.

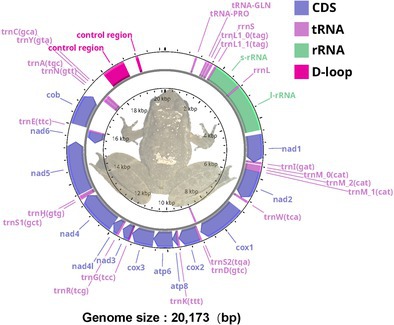

## 1 Introduction

**FIGURE 1:**
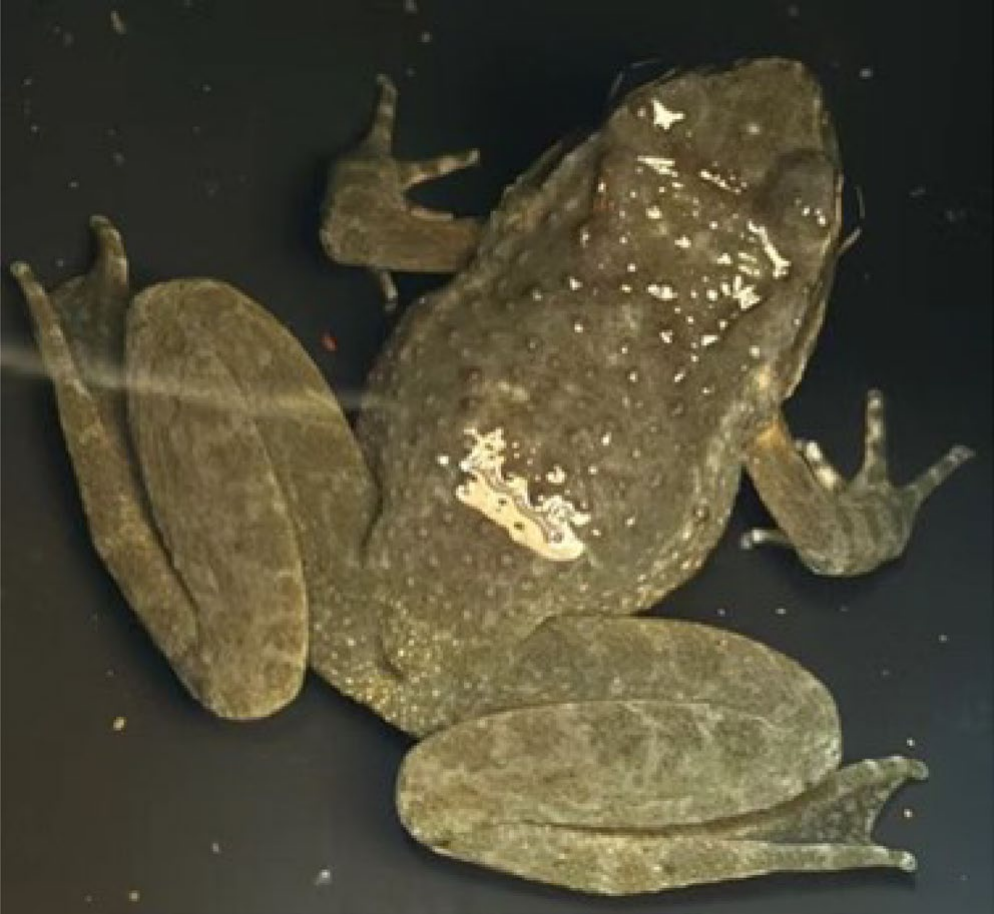
Morphological photograph of *Nanorana quadranus* (photographed by Bin Zuo at Mianyang City, Sichuan Province, China).

The genus *Nanorana* (Anura: Dicroglossidae) comprises small to medium‐sized frogs adapted to harsh high‐altitude environments. *Nanorana quadranus* , endemic to the eastern Qinghai‐Tibet Plateau, is a keystone species in alpine wetlands (Liu et al. 1960). In living specimens, distinct yellow lateral stripes are present along the body flanks; dorsal tubercles are relatively sparse and irregularly distributed (Figure 1). Due to anthropogenic landscape modification and ongoing climatic shifts, the species has been classified as Near Threatened on the IUCN Red List of Threatened Species (IUCN 2020). Although previous studies focused on its reproductive biology (Xiao et al. 2024) and taxonomic disputes with *Quasipaa* species (Chen et al. 2017), the absence of mitogenomic data hindered resolution of its evolutionary history and population genetic analysis. This study bridges this gap by presenting the first complete mitogenome of *N. quadranus* , enabling insights into its phylogenetic position.

## 2 Materials and Methods

### 2.1 Ethics Statement

All animal procedures were approved by the Yunnan University Ethics Committee (Approval No. YNU20230645) and complied with the National Wildlife Protection Law of China.

### 2.2 Sample Collection and Sequencing

An adult male *N. quadranus* specimen was collected from Qianfo Town, Mianyang City, Sichuan Province, China (latitude: 31.710, longitude: 104.262). Species identification was first conducted based on morphological characteristics following Liu et al. (1960) and regional taxonomic keys (Liu et al. 1960). In addition, the presence of a swollen vent (cloacal swelling) was compared with descriptions in the AmphibiaChina database (http://www.amphibiachina.org/species/445) to further support the taxonomic assignment (Che and Wang 2016). The specimen voucher, designated as CB237‐001R0002, is securely stored in the Ministry of Education Key Laboratory for Transboundary Ecosecurity of Southwest China at Yunnan University (Yanbo Sun, biosunyb@163.com).

Genomic DNA was extracted from the muscle tissue. Library construction was performed using the VAHTS Universal Plus DNA Library Prep Kit for Illumina (ND617). The required concentration was ≥ 1 ng/μL, with the fragment size centered around 430–530 bp and an average fragment size between 420 and 580 bp. After passing the quality check, the library was sequenced on the Illumina NovaSeq 6000 platform with a PE150 sequencing strategy.

### 2.3 De Novo Assembly and Annotation of Mitochondrial Genomes

For the raw sequencing data, we performed quality control using fastp (v0.21.0) with parameters: ‐q 10 ‐u 50 ‐y ‐g ‐Y 10 ‐e 20 ‐l 100 (S. F. Chen 2023). The mitochondrial genome sequence of *N. taihangnica* was used as a reference (Chen et al. 2014). The mitochondrial genome of *N. quadranus* was assembled using MITObim (v1.9.1) with parameters: ‐start 1 ‐end 10 ‐readpool ‐quick ‐kbait 21 (Hahn et al. 2013). After assembly, the mitochondrial genome was annotated using the MITOS (Bernt et al. 2013) and MitoFinder v1.4.2 (Allio et al. 2020). We manually merged the annotation results from both tools and performed visualization using Proksee (Grant et al. 2023). We further analyzed the tRNAs of the mitochondrial genomes of *N. quadranus* and *N. taihangnica* using tRNAscan‐SE (v.2.0.12) (Chan et al. 2021) with the parameters ‐M vert ‐thread 20. To validate the assembly reliability of the D‐loop region, we mapped the raw sequencing reads to the mitochondrial sequence using BWA v0.7.17 (Li and Durbin 2009), calculated per‐base coverage, and determined the coverage of both the D‐loop and non‐D‐loop regions.

### 2.4 Phylogenetic Analysis

Phylogenetic relationships were inferred using 13 PCGs from 29 amphibian mitogenomes (including *N. quadranus* ; Table 1). The PCGs sequences were retrieved and concatenated with FasParser software (Sun 2018). Multiple sequence alignment of each PCG was conducted in Muscle v5 (Edgar 2022). Finally, a maximum‐likelihood tree was constructed using IQ‐TREE v2.4 (‐alrt 2000 ‐bb 2000 ‐nt 40 ‐bnni) (Minh et al. 2020). To further support the taxonomic identification of our specimens as *N. quadranus* , we downloaded *cox1* (partial cds) sequences of *N. quadranus* (NCBI accession number: PP572988.1) and *N. taihangnica* (NCBI accession number: PP572987.1) from NCBI. These sequences were aligned with the full‐length *cox1* gene extracted from our newly assembled mitochondrial genome, and a phylogenetic tree was constructed using the same methods described above. In addition, pairwise genetic distances of *cox1* were calculated using the K80 (Kimura 2‐parameter) model.

**TABLE 1:** Information about the mitochondrial genome of the species used in this study.

Family	Genus	Species	Accession number	References
Dicroglossidae	*Nanorana*	*Nanorana parkeri*	NC_026789.1	(Jiang et al. 2016)
		*Nanorana pleskei*	NC_016119.1	(Chen et al. 2011)
		*Nanorana quadranus*	PV546862.1	This study
		*Nanorana taihangnica*	NC_024272.1	(Chen et al. 2014)
		*Nanorana ventripunctata* *Nanorana yunnanensis*	NC_039094.1 KF199150.2	Unpublished (Zhang et al. 2018)
	*Quasipaa*	*Quasipaa boulengeri*	NC_021937.1	(Shan et al. 2014)
		*Quasipaa exilispinosa*	NC_056269.1	(Wu et al. 2020)
		*Quasipaa robertingeri*	KY441640.1	Unpublished
		*Quasipaa spinosa*	NC_013270.1	(Zhou et al. 2009)
		*Quasipaa yei*	NC_024843.1	(Chen et al. 2015)
Ranidae	*Rana*	*Rana amurensis*	NC_030042.1	Unpublished
		*Rana chaochiaoensis*	NC_035803.1	Unpublished
		*Rana dabieshanensis*	NC_060306.1	Unpublished
		*Rana draytonii*	NC_028296.1	Unpublished
		*Rana dybowskii*	NC_023528.1	(Li, Lei, and Fu 2016)
		*Rana hanluica*	NC_061371.1	Unpublished
		*Rana huanrensis*	NC_028521.1	(Dong et al. 2016)
		*Rana jiemuxiensis*	PP797569.1	Unpublished
		*Rana johnsi*	NC_058599.1	(Chen et al. 2021)
		*Rana kukunoris*	MN733918.1	(Wang et al. 2020)
		*Rana kunyuensis*	NC_024548.1	(Li, Yin, et al. 2016)
		*Rana longicrus*	NC_061370.1	Unpublished
		*Rana omeimontis*	NC_035805.1	Unpublished
		*Rana temporaria*	NC_042226.1	(J. J. Chen 2018)
		*Rana uenoi*	NC_056272.1	Unpublished
		*Rana zhenhaiensis*	MN218687.1	(Huang et al. 2019)
		*Odorrana ishikawae*	AB511282.1	(Kurabayashi et al. 2010)
		*Lithobates sylvaticus*	NC_027236.1	(Ni et al. 2016)

## 3 Results

### 3.1 Taxonomic Identification

The phylogenetic analysis further supports that the specimens in this study belong to *N. quadranus* , as the *cox1* gene from our newly assembled mitochondrial genome clustered closely with published *N. quadranus* sequences, while *N. taihangnica* was positioned as an outgroup (Figure S1).

### 3.2 Mitogenome Organization

**FIGURE 2:**
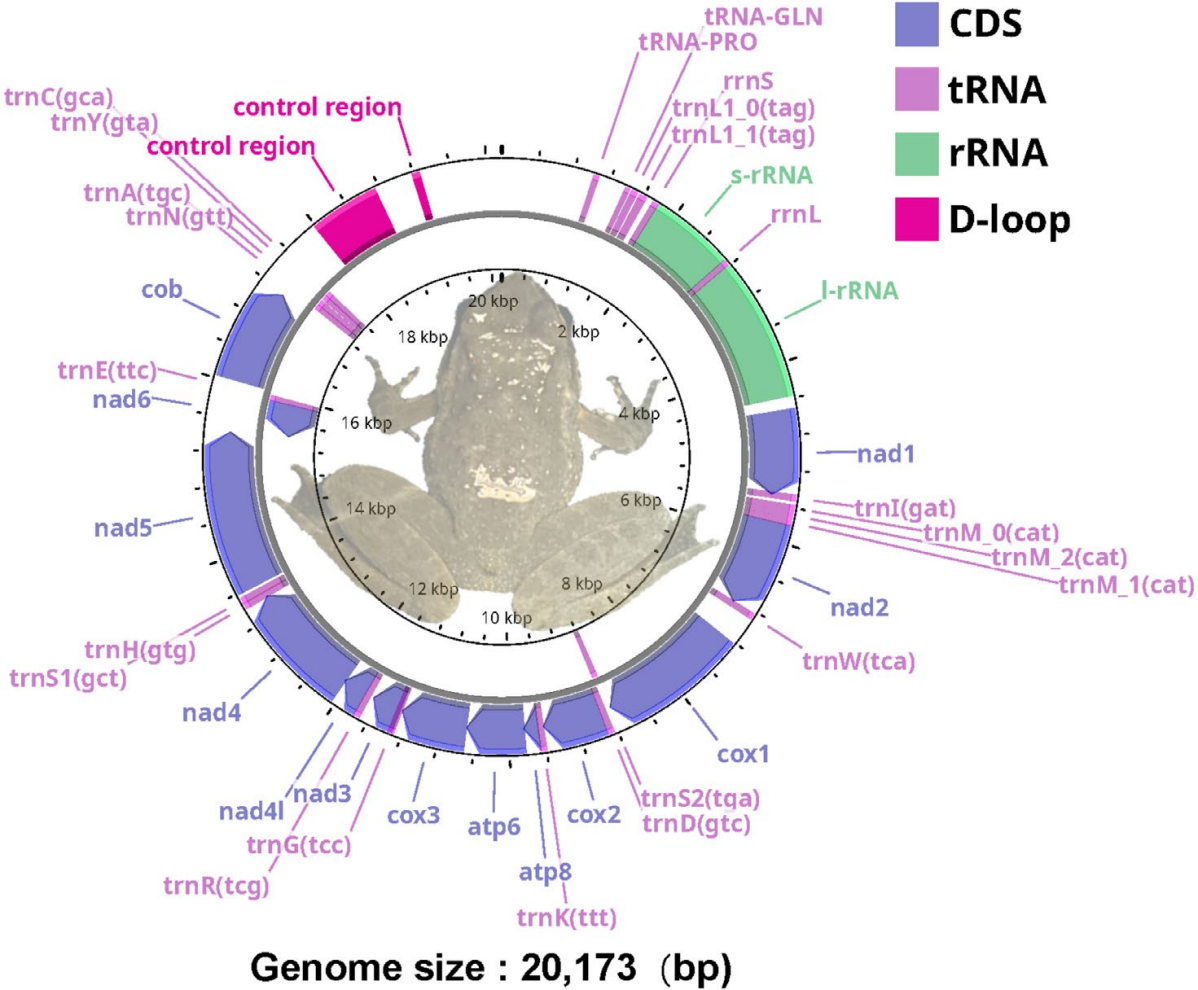
Mitochondrial genome map of the *Nanorana quadranus* . Different gene functions are represented by colored outside circles, and transcriptional direction is represented by arrows.

The mitochondrial genome of *Nanorana quadranus* showed exceptionally high sequencing quality. The coverage across the mitochondrial genome was 99.13%, with an average sequencing depth of approximately 10,310×. The mean base quality score was 37.7, and the mean mapping quality score was 55.7, indicating highly accurate base calls and reliable read alignments. The total length of the *N. quadranus* mitogenome is 20,173 bp (GenBank Accession No. PV546862), with a GC content of 41.13%, comprising 13 PCGs, 21 tRNAs (with the Threonine tRNA missing), 2 rRNAs, and one control region (Figure 2). Although the D‐loop assembly is represented by two separate intervals due to repetitive sequences, it corresponds to a single biological control region. Due to the high variability of the D‐loop region, mitochondrial genome annotation often splits a single D‐loop region into multiple segments. For example, in *Nanorana yunnanensis* (KF199150.2), three separate D‐loop regions were annotated, illustrating that the high variability of the D‐loop region poses challenges for accurate annotation. The D‐loop region shows a mean coverage of 44,692×, while the non‐D‐loop regions have a mean coverage of 8813×. Reads continuously span the entire D‐loop, supporting that the ~20 kb mitochondrial genome represents genuine biological sequence rather than an assembly artifact. Sequencing depth analysis indicates that, despite variations at the boundaries, these two annotated D‐loop regions maintain stable coverage (Figure S2). Gene order and orientation are conserved relative to other anurans (Chen et al. 2014). Twelve PCGs and 15 tRNAs are encoded on the heavy strand, while ND6 and 6 tRNAs reside on the light strand (Figure 2).

### 3.3 Phylogenetic Analysis

**FIGURE 3:**
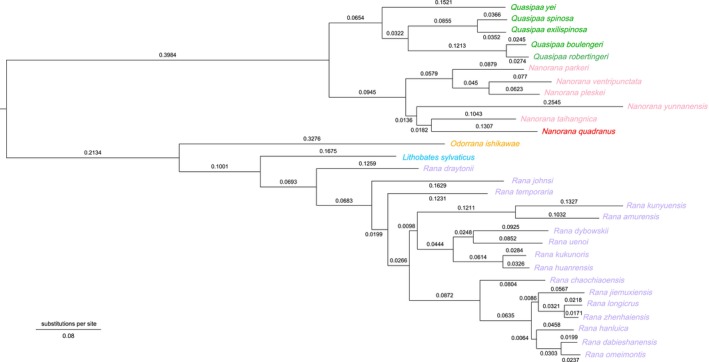
Maximum‐likelihood analysis of *N. quadranus* and 29 related species based on 13 PCG sequences with 1000 bootstraps. Branch length values (substitutions per site) are labeled near the branch. The species analyzed in this study are represented by red fonts. The sequences from the following species were used: *Nanorana parkeri* NC_026789 (Jiang et al. 2016), *Nanorana pleskei* NC_016119 (Chen et al. 2011), *Nanorana taihangnica* NC_024272 (Chen et al. 2014), *Nanorana ventripunctata* NC_039094, *Quasipaa boulengeri* NC_021937 (Shan et al. 2014), *Quasipaa exilispinosa* NC_056269 (Wu et al. 2020), *Quasipaa robertingeri* KY441640, *Quasipaa spinosa* NC_013270 (Zhou et al. 2009), *Quasipaa yei* NC_024843 (Chen et al. 2015), *Rana amurensis* NC_030042, *Rana chaochiaoensis* NC_035803, *Rana dabieshanensis* NC_060306, *Rana draytonii* NC_028296 (Li, Lei, and Fu 2016), *Rana dybowskii* NC_023528, *Rana hanluica* NC_061371, *Rana huanrensis* NC_028521 (Dong et al. 2016), *Odorrana ishikawae* AB511282 (Kurabayashi et al. 2010), *Rana jiemuxiensis* PP797569, *Rana johnsi* NC_058599 (Chen et al. 2021), *Rana kukunoris* MN733918 (Wang et al. 2020), *Rana kunyuensis* NC_024548 (Li, Yin, et al. 2016), *Rana longicrus* NC_061370, *Rana omeimontis* NC_035805, *Lithobates sylvaticus* NC_027236 (Ni et al. 2016), *Rana temporaria* NC_042226 (J. J. Chen 2018), *Rana uenoi* NC_056272, *Rana zhenhaiensis* MN218687 (Huang et al. 2019), *Nanorana yunnanensis* KF199150.2 (Zhang et al. 2018).

The phylogenetic tree (Figure 3) robustly supports the monophyly of Dicroglossidae (Bootstrap support [BS] = 100%). *N. quadranus* clusters with *N. taihangnica* (BS = 100%), aligning with previous classifications (Chen et al. 2017).

## 4 Discussion and Conclusion

The assembly of the first complete mitogenome of *N. quadranus* provides critical insights into the evolutionary relationships of this high‐altitude endemic species and lays a foundation for future studies on high‐altitude adaptations, particularly involving mitochondrial genes related to energy metabolism. Notably, the close phylogenetic clustering of *N. quadranus* with *N. taihangnica* (BS = 100%) robustly supports its taxonomic placement within the Dicroglossidae family. Additionally, integrating nuclear genomic data, such as single‐copy orthologs or sex‐linked markers (Xiao et al. 2024), would refine phylogenetic hypotheses and elucidate whether adaptive traits (e.g., cold tolerance) originated via mito‐nuclear coevolution or independent nuclear mutations.

Given the close phylogenetic relationship between *N. quadranus* and *N. taihangnica* , we conducted additional analyses to clarify their taxonomic distinction. Specifically, we reconstructed a phylogenetic tree using the published *cox1* sequences of both species together with the *cox1* sequence generated in this study. The results robustly placed our specimen within *N. quadranus* and clearly differentiated *N. quadranus* from *N. taihangnica* . Furthermore, diagnostic morphological characters also support this conclusion: *N. quadranus* exhibits a square‐shaped cutaneous swelling surrounding the cloaca, a feature absent in *N. taihangnica* . This morphological distinction is consistent with the species description provided in the AmphibiaChina database (Che and Wang 2016), thereby reinforcing the recognition of *N. quadranus* as a species distinct from *N. taihangnica* . Our analysis shows that the new *N. quadranus cox1* differs from the published partial fragment by ~5%, while both differ from *N. taihangnica cox1* by ~18%, supporting them as distinct species.

Based on the phylogenetic analysis, *N. yunnanensis* clustered with *N. quadranus* and *N. taihangnica* , which further supports the previously proposed view that the genus *Feirana* is invalid (Zhang et al. 2018).

Regarding the loss of tRNA‐thr in our assembly, we carefully examined the annotation and compared it with closely related species and found that the loss of tRNA‐thr is not due to a software error but represents a branch‐specific gene loss. In the mitochondrial genome of *N. taihangnica* , a close relative of *N. quadranus* , tRNA‐thr was also not identified (Figure S3), suggesting an ancestral loss resulting from a certain evolutionary event. Combined with tRNAscan‐SE analysis of the complete mitochondrial genomes of both species (Tables S1 and S2), the results further support the conclusion of a lineage‐specific loss rather than an artifact of assembly or software error. Although the loss or duplication of tRNAs is relatively rare in vertebrates, previous studies have documented tRNA gene loss and rearrangements in the mitochondrial genomes of vertebrates such as Gekkonidae (Kumazawa et al. 2014), representing a branch‐specific evolutionary event.

## Author Contributions

**Jia Liu:** data curation (equal), investigation (equal), methodology (equal), software (equal), writing – original draft (equal). **Bin Zuo:** data curation (equal), investigation (equal), methodology (equal), visualization (equal), writing – original draft (equal). **Yan‐Bo Sun:** conceptualization (lead), funding acquisition (lead), project administration (lead).

## Funding

This work was supported by the National Key Research Development Program of China (2022YFF0802300) and the Yunnan Fundamental Research Projects (202401BC070011).

## Disclosure

The authors have nothing to report.

## Ethics Statement

This study involved research on *Nanorana quadranus* , a Near Threatened species classified by the International Union for Conservation of Nature (IUCN). All procedures strictly adhered to the IUCN Guidelines for Appropriate Uses of Red List Data. We further complied with the principles of the Convention on Biological Diversity (CBD) regarding access to genetic resources and benefit‐sharing, and confirmed that no activities related to international trade of specimens (as regulated by CITES) were conducted. Ethical approval for this research was granted by the Yunnan University Ethics Committee (YNU20230645), and all data collection protocols were designed to minimize disturbance to wild populations.

## Conflicts of Interest

The authors declare no conflicts of interest.

## Supporting information


**Figure S1:** The phylogenetic analysis further supports that the specimens in this study belong to *N. quadranus* , as the *cox1* gene from our newly assembled mitochondrial genome clustered closely with published *N. quadranus* sequences, while *N. taihangnica* was positioned as an outgroup.
**Figure S2:**. Analysis of sequencing depth in the D‐loop regions. The x‐axis represents the nucleotide positions within the D‐loop regions, and the y‐axis shows the sequencing depth on a log10 scale.
**Figure S3:**. Complete mitochondrial genome of *N. taihangnica* (NCBI Reference Sequence: NC_024272.1) and its visualization. The result shows that the mitochondrial genome of *N. taihangnica* lacks the tRNA‐Thr.
**Table S1:**. Results of tRNAscan‐SE analysis of the mitochondrial genome of *N. quadranus* .
**Table S2:**. Results of tRNAscan‐SE analysis of the mitochondrial genome of *N. taihangnica* .

## Data Availability

The genome sequence data that support the findings of this study are openly available in GenBank of NCBI at (https://www.ncbi.nlm.nih.gov/) under the accession no. PV546862. The associated BioProject, SRA, and Bio‐Sample numbers are PRJNA1254796, SRR33295323, and SAMN48124507, respectively.
