# Supplementary material for: The Complete Mitochondrial Genome of Nearly Threatened Kwang‐Yang Asian Frog Nanorana quadranus (Anura: Dicroglossidae) and Its Phylogenetic Analyses
Source: Ecol Evol. 2026 Jan 11;16(1):e72877. doi: 10.1002/ece3.72877 (PMC12790872; doi:10.1002/ece3.72877)
Supplement: Supplementary file 1 — Figure S1: The phylogenetic analysis further supports that the specimens in this study belong to N. quadranus , as the cox1 gene from our newly assembled mitochondrial genome clustered closely with published N. quadranus sequences, while N. taihangnica was positioned as an outgroup. Figure S2:. Analysis of sequencing depth in the D‐loop regions. The x‐axis represents the nucleotide positions within the D‐loop regions, and the y‐axis shows the sequencing depth on a log10 scale. Figure S3:. Complete mitochondrial genome of N. taihangnica (NCBI Reference Sequence: NC_024272.1) and its visualization. The result shows that the mitochondrial genome of N. taihangnica lacks the tRNA‐Thr. Table S1:. Results of tRNAscan‐SE analysis of the mitochondrial genome of N. quadranus . Table S2:. Results of tRNAscan‐SE analysis of the mitochondrial genome of N. taihangnica . [file ECE3-16-e72877-s001.docx]

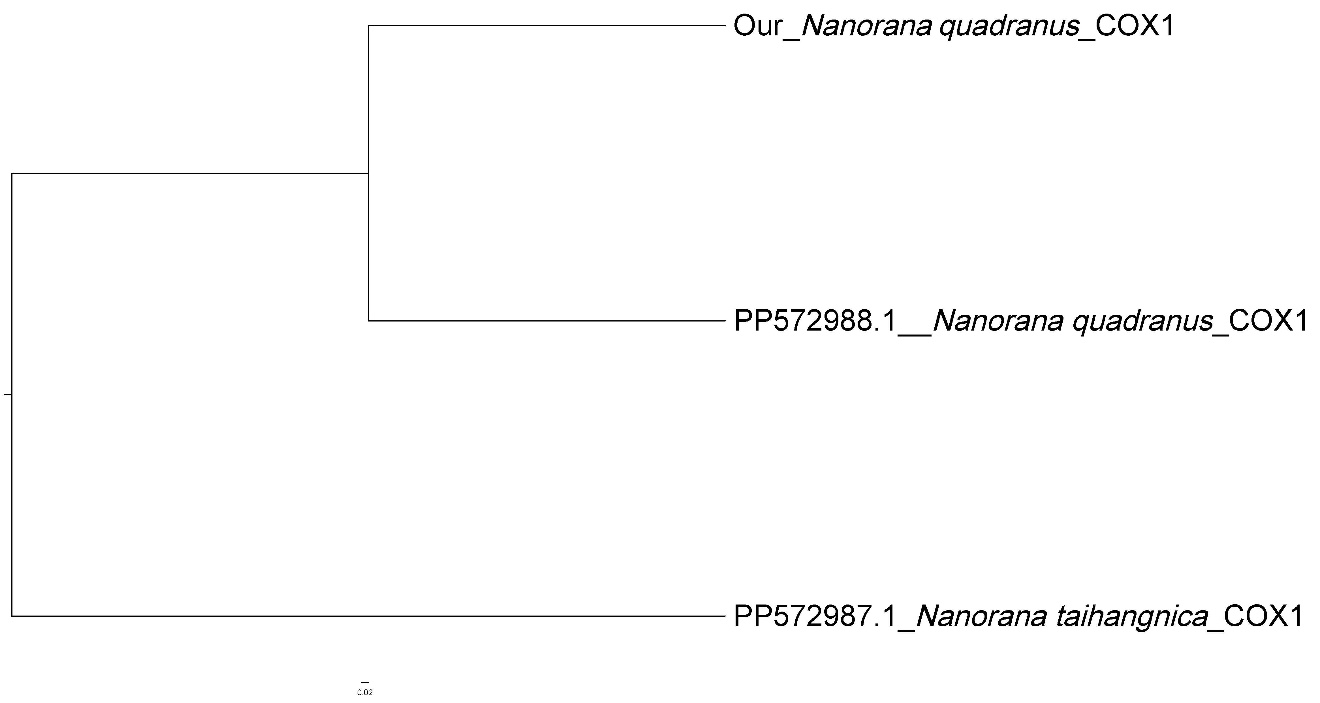


**Figure S1.** The phylogenetic analysis further supports that the specimens in this study belong to *N. quadranus*, as the *cox1* gene from our newly assembled mitochondrial genome clustered closely with published *N. quadranus* sequences, while *N. taihangnica* was positioned as an outgroup.


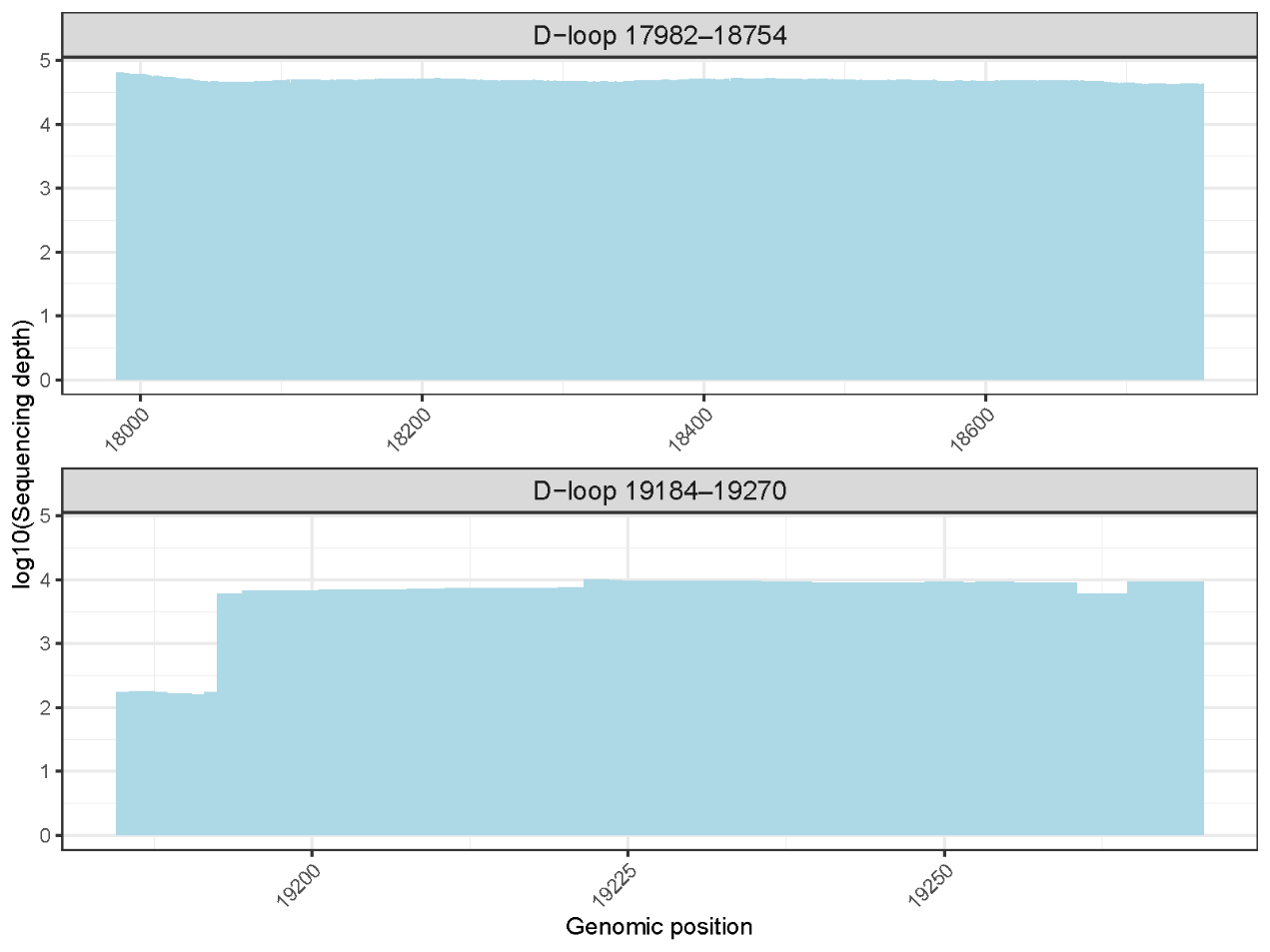


**Figure S2. Analysis of sequencing depth in the D-loop regions.** The x-axis represents the nucleotide positions within the D-loop regions, and the y-axis shows the sequencing depth on a log10 scale.


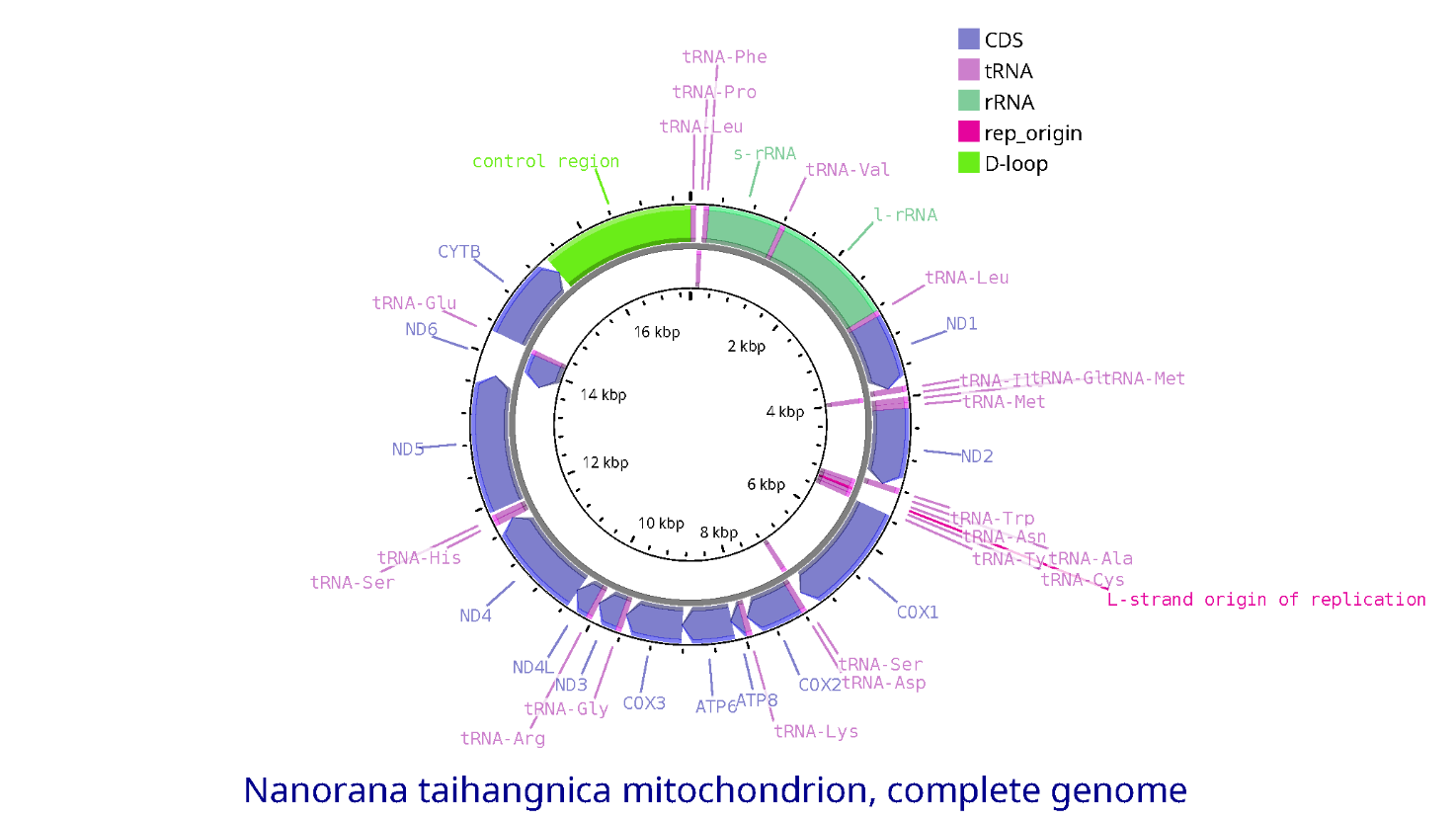


**Figure S3.** Complete mitochondrial genome of *N. taihangnica* (NCBI Reference Sequence: NC_024272.1) and its visualization. The result shows that the mitochondrial genome of *N. taihangnica* lacks the tRNA-Thr.

| Sequence |  | tRNA | Bounds | tRNA | Anti | Intron Bounds | Inf |  |
| --- | --- | --- | --- | --- | --- | --- | --- | --- |
| Name | tRNA # | Begin | End | Type | Codon | Begin | End | Score |
| -------- | ------ | ----- | ------ | ---- | ----- | ----- | ---- | ------ |
| PV546862.1 | 1 | 1456 | 1527 | Leu | TAG | 0 | 0 | 78.2 |
| PV546862.1 | 2 | 1715 | 1782 | Phe | GAA | 0 | 0 | 57.0 |
| PV546862.1 | 3 | 2716 | 2785 | Val | TAC | 0 | 0 | 75.6 |
| PV546862.1 | 4 | 5455 | 5525 | Ile | GAT | 0 | 0 | 74.0 |
| PV546862.1 | 5 | 5567 | 5635 | Met | CAT | 0 | 0 | 92.0 |
| PV546862.1 | 6 | 5646 | 5714 | Met | CAT | 0 | 0 | 79.8 |
| PV546862.1 | 7 | 5725 | 5793 | Met | CAT | 0 | 0 | 74.5 |
| PV546862.1 | 8 | 6827 | 6896 | Trp | TCA | 0 | 0 | 93.0 |
| PV546862.1 | 9 | 8837 | 8905 | Asp | GTC | 0 | 0 | 76.1 |
| PV546862.1 | 10 | 9593 | 9662 | Lys | TTT | 0 | 0 | 80.6 |
| PV546862.1 | 11 | 11286 | 11354 | Gly | TCC | 0 | 0 | 95.3 |
| PV546862.1 | 12 | 11695 | 11763 | Arg | TCG | 0 | 0 | 72.6 |
| PV546862.1 | 13 | 13406 | 13474 | His | GTG | 0 | 0 | 84.8 |
| PV546862.1 | 14 | 13475 | 13542 | Ser | GCT | 0 | 0 | 37.6 |
| PV546862.1 | 15 | 17581 | 17516 | Tyr | GTA | 0 | 0 | 84.2 |
| PV546862.1 | 16 | 17514 | 17450 | Cys | GCA | 0 | 0 | 68.6 |
| PV546862.1 | 17 | 17429 | 17357 | Asn | GTT | 0 | 0 | 93.6 |
| PV546862.1 | 18 | 17355 | 17287 | Ala | TGC | 0 | 0 | 52.9 |
| PV546862.1 | 19 | 15966 | 15903 | Glu | TTC | 0 | 0 | 51.4 |
| PV546862.1 | 20 | 8836 | 8766 | Ser | TGA | 0 | 0 | 94.3 |
| PV546862.1 | 21 | 1077 | 1014 | Pro | TGG | 0 | 0 | 15.8 |

**Table S1. Results of tRNAscan-SE analysis of the mitochondrial genome of *N. quadranus*.**

| Sequence |  | tRNA | Bounds | tRNA | Anti | Intron Bounds | Inf |  |
| --- | --- | --- | --- | --- | --- | --- | --- | --- |
| Name | tRNA # | Begin | End | Type | Codon | Begin | End | Score |
| -------- | ------ | ----- | ------ | ---- | ----- | ----- | ---- | ------ |
| NC_024272.1 | 1 | 1 | 72 | Leu | TAG | 0 | 0 | 68.3 |
| NC_024272.1 | 2 | 176 | 240 | Phe | GAA | 0 | 0 | 16.4 |
| NC_024272.1 | 3 | 1176 | 1245 | Val | TAC | 0 | 0 | 68.5 |
| NC_024272.1 | 4 | 2832 | 2902 | Leu | TAA | 0 | 0 | 56.6 |
| NC_024272.1 | 5 | 3861 | 3931 | Ile | GAT | 0 | 0 | 75.4 |
| NC_024272.1 | 6 | 4002 | 4070 | Met | CAT | 0 | 0 | 98.2 |
| NC_024272.1 | 7 | 4077 | 4145 | Met | CAT | 0 | 0 | 80.4 |
| NC_024272.1 | 8 | 5179 | 5248 | Trp | TCA | 0 | 0 | 96.6 |
| NC_024272.1 | 9 | 7159 | 7226 | Asp | GTC | 0 | 0 | 90.9 |
| NC_024272.1 | 10 | 7914 | 7983 | Lys | TTT | 0 | 0 | 83.4 |
| NC_024272.1 | 11 | 9607 | 9675 | Gly | TCC | 0 | 0 | 98.1 |
| NC_024272.1 | 12 | 10016 | 10084 | Arg | TCG | 0 | 0 | 77.7 |
| NC_024272.1 | 13 | 11727 | 11795 | His | GTG | 0 | 0 | 91.1 |
| NC_024272.1 | 14 | 11771 | 11863 | Ser | GCT | 0 | 0 | 39.2 |
| NC_024272.1 | 15 | 14288 | 14220 | Glu | TTC | 0 | 0 | 87.5 |
| NC_024272.1 | 16 | 7158 | 7088 | Ser | TGA | 0 | 0 | 93.9 |
| NC_024272.1 | 17 | 5541 | 5474 | Tyr | GTA | 0 | 0 | 76.8 |
| NC_024272.1 | 18 | 4001 | 3931 | Gln | TTG | 0 | 0 | 94.6 |
| NC_024272.1 | 19 | 176 | 108 | Pro | TGG | 0 | 0 | 67.2 |

**Table S2. Results of tRNAscan-SE analysis of the mitochondrial genome of *N. taihangnica*.**
